# Technological Approaches for Olive Oil Flavoring and Their Impact on Oxidative Stability and Nutritional Quality: A Review

**DOI:** 10.3390/molecules31193507

**Published:** 2026-10-01

**Authors:** Hiba Rahmani, Hammadi El Farissi, Fatima-Zahra Azar, Karim Danoun, Francesco Cacciola, Achraf El Kasmi, Yahya El Hammoudani, Fouad Dimane

**Affiliations:** 1 Chemical Engineering for Resources Valorization Group (UAE/L01FST), Faculty of Science and Technology, Abdelmalek Essaadi University, Tangier 90002, Morocco; rahmani.hiba@etu.uae.ac.ma; 2 Department of Chemical and Biochemical Sciences (CBS), College of Chemical Sciences and Engineering (CCSE), Mohammed VI Polytechnic University (UM6P), Benguerir 43150, Morocco; f.azar@uae.ac.ma (F.-Z.A.); karim.danoun@um6p.ma (K.D.); 3 Laboratory of Engineering Sciences and Applications (LSIA), National School of Applied Sciences (ENSAH), Abdelmalek Essaadi University, Al-Hoceima 32003, Morocco; a.elkasmi@uae.ac.ma (A.E.K.); y.elhammoudani@uae.ac.ma (Y.E.H.); f.dimane@uae.ac.ma (F.D.); 4 Messina Institute of Technology c/o Department of Chemical, Biological, Pharmaceutical and Environmental Sciences, University of Messina, Viale G. Palatucci 13, 98168 Messina, Italy

**Keywords:** flavored olive oil, phenolic compounds, volatile compounds, oxidative stability, ultrasound, pulsed electric field, microwave extraction, high-pressure processing, supercritical CO_2_ extraction

## Abstract

Olive oil flavoring is gaining attention as a means of improving sensory characteristics, nutritional quality, and oxidative stability. However, current evidence remains fragmented across botanical ingredients, operating conditions, and production methods. This review critically compares conventional approaches, including maceration, infusion, co-processing, and direct addition, with emerging processing strategies. Particular attention is given to the transfer of phenolic and volatile compounds, extraction performance, oil quality, energy demand, scalability, economic viability, and regulatory considerations. Conventional methods are generally accessible and cost-effective, but extended contact times and the use of fresh plant materials may increase acidity and peroxide value. Emerging technologies can accelerate and improve the control of mass transfer while promoting the recovery or retention of bioactive and aroma compounds. Nevertheless, their performance varies with the plant matrix, olive cultivar, operational parameters, and production scale. Wider commercial application is also constrained by limited standardization, substantial equipment costs, insufficient long-term evidence, and uncertain regulatory frameworks. Future studies should develop comparable protocols, explore combined processing strategies, evaluate product quality and safety during storage, and conduct robust techno-economic assessments. Validation under pilot- and full-scale conditions in diverse geographical and regulatory settings will be essential to support the safe, consistent, and economically viable production of flavored olive oils.

## 1. Introduction

*Olea europaea* L., a member of the Oleaceae family, has been cultivated for thousands of years for both olive oil production and medicinal purposes, particularly in Mediterranean regions characterized by warm-temperate and subtropical climates [1]. The olive tree is believed to have originated in ancient Persia and Mesopotamia approximately 5000 years ago before spreading throughout the Mediterranean Basin, where it became an agricultural and cultural symbol. Today, countries including Spain, Portugal, Italy, Greece, Turkey, and Morocco are among the world’s leading olive oil producers [2]. According to the most recent statistical report of the International Olive Council, global olive oil production reached approximately 3.57 million tonnes during the 2024/2025 crop year, underscoring the considerable economic importance of the sector and the continued predominance of Mediterranean-producing countries [3].

The flavoring of olive oil represents the continuation of a long-standing culinary tradition in which oils acquire the aroma and bioactive constituents of herbs, spices, fruits, and other plant materials. In addition to enhancing sensory characteristics, flavoring may increase the nutritional value and shelf life of olive oil by enriching it with antioxidant and antimicrobial compounds derived from the added ingredients [4]. Recent studies have shown that the incorporation or co-processing of plant materials can modify the phenolic and volatile profiles of olive oil, resulting in products with distinctive sensory and functional properties [5]. In recent years, scientific interest in flavored olive oils has increased considerably, driven in part by advances in extraction and processing technologies that enable more efficient and controlled incorporation of bioactive and aroma compounds. Consequently, olive oil flavoring has evolved from a traditional culinary practice into an emerging area of food science and processing research.

The quality, nutritional value, and oxidative stability of extra virgin olive oil (EVOO) are largely determined by its chemical composition. Its minor constituents include phenolic compounds, such as secoiridoids, phenolic alcohols, phenolic acids, flavonoids, and lignans, which contribute significantly to antioxidant activity and health-promoting properties [6,7]. Among these constituents, hydroxytyrosol, tyrosol, oleocanthal, and oleacein have received particular attention because of their antioxidant, anti-inflammatory, and other biological activities, which contribute to the functional properties associated with olive oil consumption [8,9,10,11]. In addition, volatile compounds generated primarily through the lipoxygenase pathway are responsible for the characteristic aroma and flavor of high-quality EVOO [12]. The final chemical profile of olive oil is influenced by several agronomic factors, including cultivar, fruit maturity, and climatic conditions, as well as technological parameters such as milling, malaxation, extraction, and storage [13]. These factors affect both the concentration and preservation of antioxidant compounds and, consequently, the susceptibility of the oil to oxidative deterioration. The incorporation of flavoring materials may further modify this complex chemical matrix by introducing additional phenolic and volatile compounds. The resulting effects on oxidative stability and nutritional quality depend on the botanical source, concentration, processing conditions, and chemical composition of the added material [5].

In an effort to improve product quality while reducing processing time and enhancing environmental and economic sustainability, several innovative technologies have been investigated, including pulsed electric field (PEF), high-pressure processing (HPP), ultrasound-assisted extraction (UAE), microwave-assisted extraction (MAE), and supercritical CO_2_ extraction (sc-CO_2_). These approaches can enhance mass transfer, facilitate the recovery or incorporation of bioactive compounds, and shorten processing times; however, their performance is highly dependent on the characteristics of the raw material and the selected operating conditions. Despite their demonstrated potential to improve extraction efficiency and preserve bioactive compounds, their application to the production of flavored EVOO remains relatively limited. In particular, industrial-scale evidence specifically addressing flavored olive oils is considerably scarcer than that available for conventional olive oil processing or for the extraction of bioactive compounds from olive-derived matrices. Moreover, consumer acceptance remains an important consideration, as concerns regarding novel food-processing technologies may influence their market uptake [14]. So far, most investigations on flavored olive oils have focused on individual flavoring material or specific processing technique, making direct comparisons difficult and hindering the establishment of standardized production protocols. Previous reviews have examined selected aspects of this field, including olive oil co-extraction, conventional and innovative malaxation technologies, emerging technologies for virgin olive oil processing, the enrichment of vegetable oils with plant-derived antioxidants, and individual advanced extraction approaches [7,12,15,16,17]. However, these topics have generally been considered separately. A comprehensive assessment integrating flavoring technologies with phenolic and volatile compound transfer, oxidative stability, nutritional quality, processing efficiency, energy consumption, scalability, economic feasibility, and regulatory requirements is still lacking.

Accordingly, the present review provides an integrated and comparative assessment of conventional and emerging technologies for olive oil flavoring within a common analytical and technological framework. Particular attention is given to (i) elucidating the mechanisms governing the transfer of phenolic and volatile compounds from flavoring materials into the oil matrix; (ii) critically assessing the effects of different flavoring techniques on oxidative stability, chemical composition, and nutritional quality based on quantitative evidence; (iii) comparing processing efficiency, energy requirements, scalability, economic feasibility, and regulatory constraints among the different technologies; (iv) identifying methodological limitations and the lack of protocol standardization that hinder comparisons among published studies; and (v) proposing future research priorities, particularly regarding industrial-scale implementation, process optimization, long-term stability, safety assessment, and regulatory harmonization. Through this integrated perspective, the review aims to identify the most promising technological approaches for the development of high-quality, stable, safe, and commercially viable flavored olive oils.

## 2. Chemical Composition of Olive Oil

The chemical composition of extra virgin olive oil (EVOO) is determined by a combination of agronomic and technological factors, including cultivar, fruit maturity, health status, climatic conditions, crushing, malaxation, extraction, filtration, and storage [13]. As summarized in Table 1, triacylglycerols account for approximately 98–99% of EVOO and are predominantly composed of oleic, palmitic, and linoleic acids, whereas the minor fraction comprises free fatty acids, sterols, tocopherols, pigments, volatile compounds, waxes, and phenolic constituents. Oleic acid is the predominant fatty acid and contributes to the relatively high oxidative resistance of olive oil, whereas higher proportions of polyunsaturated fatty acids increase its susceptibility to oxidation. Among the minor components, phenolic compounds are particularly important because they contribute to antioxidant activity, nutritional value, and sensory properties [6,9].

They are generally classified into secoiridoids, phenolic alcohols, phenolic acids, flavonoids, and lignans [7], including compounds such as hydroxytyrosol, tyrosol, oleuropein, ligstroside, oleacein, oleocanthal, luteolin, apigenin, pinoresinol, and 1-acetoxypinoresinol. Tocopherols, mainly α-tocopherol, together with chlorophylls, carotenoids, phytosterols such as β-sitosterol, and volatile compounds, also contribute to the nutritional, sensory, and oxidative characteristics of EVOO. The concentrations of these bioactive constituents are strongly influenced by fruit ripening and storage conditions. Advanced fruit maturity and delayed processing generally result in losses of phenolic compounds, tocopherols, and pigments, thereby lowering antioxidant capacity and oxidative stability [13].

Lipid oxidation is one of the principal causes of EVOO deterioration because it affects its chemical composition, sensory quality, and nutritional value. Oxidative processes involve the formation of lipid radicals, including alkyl radicals, which subsequently react with molecular oxygen to generate peroxyl radicals and ultimately lipid hydroperoxides as primary oxidation products. The peroxide value (PV) is therefore commonly used to assess primary oxidation, while K232 and K270 are used to monitor conjugated dienes and trienes associated with oxidative deterioration [18,19].

Flavoring can further modify the chemical balance of EVOO by introducing additional phenolic and volatile compounds into the oil matrix. Depending on the botanical ingredient, its concentration, contact time, and processing method, these additions may either enhance or reduce oxidative stability. For example, in flavored ‘Madural’ EVOOs, the concentration of phenolic compounds and tocopherols varied according to the flavoring agent, whereas the characteristic fatty-acid profile remained largely unchanged. Similarly, Chahdoura et al. [20] reported that the flavoring method significantly affected physicochemical quality and oxidative behavior, although oleic acid (C18:1) remained the predominant fatty acid, followed by linoleic acid (C18:2n-6) and palmitic acid (C16:0). These findings suggest that flavoring primarily modifies the minor bioactive and volatile fractions of olive oil rather than substantially altering its major lipid composition.

However, the effects of flavoring on oxidative stability cannot be predicted solely from changes in total phenolic content. The final oxidative behavior also depends on the chemical nature and concentration of the transferred phenolic compounds, their structural characteristics and interactions with other constituents, their distribution within the lipid matrix, and the possible presence of pro-oxidant components. Consequently, an increase in total phenolic content does not necessarily result in a proportional improvement in oxidative stability [21,22,23,24].

## 3. Volatile Compounds of EVOO

EVOO contains a complex volatile fraction that plays a central role in its characteristic aroma, sensory quality, and consumer acceptance. Guerfel et al. [25] reported that the volatile fraction of virgin olive oil (VOO) consists mainly of aldehydes, ketones, alcohols, and esters, with C6 compounds being particularly important contributors to green and fresh aroma notes. Similarly, Tanouti et al. [26,27] identified 84 volatile compounds in virgin olive oils from eastern Morocco by SPME-GC/MS, belonging mainly to the classes of aldehydes, alcohols, esters, ketones, carboxylic acids, and hydrocarbons. The main classes of volatile compounds, representative molecules, sensory relevance, formation pathways, and the principal factors affecting their concentrations are summarized in Table 2. According to Angerosa et al. [28] and Aparicio and Morales [29] many of the volatile compounds responsible for the desirable aroma of VOO originate from the lipoxygenase (LOX) pathway, which is activated during fruit crushing and malaxation. In this pathway, polyunsaturated fatty acids undergo enzymatic oxidation and subsequent cleavage, leading to the formation of C6 aldehydes, which can subsequently be reduced to the corresponding alcohols and esterified to form esters. These compounds contribute differently to the characteristic sensory attributes of olive oil. In particular, hexanal, trans-2-hexenal, trans-2-hexen-1-ol, and cis-3-hexenyl acetate are associated with green, fresh, and fruity notes, whereas C5 compounds such as 1-penten-3-one may contribute to other characteristic sensory attributes, including pungent and green notes. The relative contribution of individual volatiles to sensory perception is strongly dependent on their concentration, odor thresholds, and interactions within the complex olive oil matrix.

The volatile profile is also strongly influenced by cultivar and fruit maturity. Aparicio and Morales [29] demonstrated clear varietal and ripening-related changes in green-aroma compounds in oils from Arbequina, Picual, Koroneiki, and Coratina cultivars. Similarly, Cecchi and Alfei [30] confirmed that genetic factors significantly affect volatile formation and suggested that terpene hydrocarbons may serve as useful markers of cultivar and geographical origin.

**Table 2:** Major Volatile Compounds of EVOO, their origin and sensory relevance.

Chemical Class	Representative Volatile Compounds	Origin/Formation	Reported Sensory Relevance	Main Factors Affecting Concentration	Ref
C6 aldehydes	Hexanal, (E)-2-hexenal, (Z)-3-hexenal, (E)-3-hexenal	LOX pathway from linoleic and α-linolenic acids; hydroperoxides are cleaved by hydroperoxide lyase	Important contributors to green and fruity notes; (E)-2-hexenal is associated with cut-grass/lawn and bitter-almond perceptions; hexanal contributes to green and other sensory attributes	Cultivar, ripeness, malaxation time and temperature, agronomic conditions	[26,28,29,31]
C6 alcohols	Hexan-1-ol, (Z)-3-hexen-1-ol, (E)-3-hexen-1-ol, (E)-2-hexen-1-ol	Reduction of C6 aldehydes by alcohol dehydrogenase (ADH)	(Z)-3-hexen-1-ol is associated with green sensations; some C6 alcohols can be associated with less attractive sensory perceptions depending on their concentration	Cultivar/ADH activity, ripening, malaxation temperature and duration	[25,28,29]
C6 esters	Hexyl acetate, (Z)-3-hexenyl acetate	Esterification of C6 alcohols by alcohol acetyltransferase	Important contributors to delicate green, fruity and banana-like notes	Malaxation time and particularly temperature; cultivar/AAT activity	
C5 carbonyls and alcohols	1-Penten-3-one, 1-penten-3-ol, (E)-2-pentenal, (E)-2-penten-1-ol	Secondary reactions associated with the LOX pathway	1-Penten-3-one contributes to several sensory attributes and has been related to bitter and pungent perceptions	Malaxation temperature and duration; cultivar	[28]
Terpenes/hydrocarbons	3-Carene, terpene hydrocarbons and other cultivar-dependent hydrocarbons	Mainly related to genetic and varietal characteristics	Can contribute to varietal aroma and may serve as markers of cultivar or geographic origin	Genotype, cultivar, geographical origin	[30]
Short-chain esters associated with defects	Ethyl butanoate, ethyl propanoate, butyl acetate	Mainly associated with fermentative processes in inadequately stored fruits	Ethyl butanoate showed particularly high sensory relevance in the fusty defect	Pre-extraction fruit storage and microbial activity	[31]
Short-chain acids	Acetic, propanoic, butanoic, pentanoic acids	Fermentative metabolism and/or degradation reactions	Acetic acid is strongly associated with winey–vinegary notes; propanoic and butanoic acids contribute to fusty defects	Fruit storage, fermentation and microbial activity	
Microbial/defect-related alcohols	3-Methylbutan-1-ol, 1-octen-3-ol	Microbial degradation and fermentation	1-Octen-3-ol is a major contributor to mustiness–humidity; 3-methylbutanol contributes to winey–vinegary and other defective profiles	Poor fruit storage, humidity and microbial development	
Oxidation-derived aldehydes	Nonanal, (E)-2-heptenal, (E)-2-octenal, 2,4-heptadienal, 2,4-decadienal	Secondary products of lipid autoxidation	Fatty, oily, painty, pungent and rancid notes	Oxygen exposure, storage time and oxidative deterioration	[31,32]
Storage-related VOCs	Hexanal, nonanal, trans,trans-2,4-octadienal, acetic acid, 1-octen-3-ol	Increase during progressive oxidative/storage deterioration	Associated with reduction in fresh/herbaceous sensory characteristics during use	Time, oxygen exposure after bottle opening, initial phenolic content	[32]

Processing and storage conditions further determine the final volatile profile of VOO. Angerosa et al. [28] showed that prolonged malaxation increased the concentrations of several C6 and C5 aldehydes and alcohols, whereas C6 esters, particularly cis-3-hexenyl acetate, decreased. Increasing the malaxation temperature also resulted in reductions in several desirable green volatiles. Their experiments, conducted at 25 and 35 °C for 15–90 min, indicated that relatively low temperatures and malaxation times of approximately 30–45 min provided a suitable compromise between extraction yield and preservation of sensory quality. Servili et al. [33] demonstrated that the oxygen concentration in the malaxer headspace strongly affected the phenolic composition of the resulting oil, whereas the formation of LOX-derived volatile compounds was comparatively less affected.

During storage, however, oxidative and, under inappropriate conditions, microbial processes can contribute to the formation of undesirable volatile compounds and the development of sensory defects. Morales [31] associated 1-octen-3-ol with musty–humid odors; ethyl butanoate and short-chain fatty acids with fusty defects; acetic acid, 3-methylbutanol, and ethyl acetate with winey–vinegary defects; and several saturated and unsaturated aldehydes and acids with rancid notes. More recently, Donnarumma [32] observed that one month of simulated domestic consumption increased oxidation-related compounds, including hexanal, nonanal, trans-2,4-octadienal, acetic acid, and 1-octen-3-ol, accompanied by a progressive loss of herbaceous sensory notes.

Collectively, the available studies indicate that the volatile profile of EVOO result from interactions among cultivar, fruit maturity, enzymatic activity, processing conditions, oxygen availability, and storage [28,29,30,31,32,33]. The preservation of LOX-derived green and fruity volatiles therefore requires careful control of both pre-harvest and processing variables. In flavored olive oils, the addition or co-processing of botanical materials may introduce exogenous volatile compounds and modify the release, partitioning, and perception of endogenous olive-oil volatiles, thereby affecting the sensory balance and stability of the final product [20,32].

## 4. Flavoring Technologies

### 4.1. Conventional Extraction Technologies

#### 4.1.1. Maceration and Infusion

Maceration and infusion are conventional flavoring methods based on prolonged contact between olive oil and fresh or dried botanical materials, generally at room temperature or under mild heating [34]. These approaches are relatively simple, require limited technological equipment, and allow the gradual transfer of volatile and non-volatile compounds from the plant material to the oil matrix. However, prolonged contact may increase the risk of oxidative or hydrolytic deterioration, particularly when fresh botanical materials with relatively high moisture contents are used [35,36].

Custureri et al. [35] reported that infusion with 2% freeze-dried bergamot for 30 days resulted in a total phenolic content of 457.80 mg GAE/kg after 360 days of storage. Nevertheless, the peroxide value reached 16.73 mEq O_2_/kg, close to the 17.89 mEq O_2_/kg measured in the unflavored control, suggesting that the treatment provided limited protection against primary oxidation over prolonged storage. Caponio et al. [36] reported that conventional infusion required 7–15 days at 15–18 °C, whereas combined malaxation required only 30 min at 2 °C. Infused oils also generally showed greater oxidative degradation. In the same study, total phenolic contents of basil-, chili-, and chili–garlic-infused oils were 373, 391, and 380 mg GAE/kg, respectively, while DPPH antioxidant activity ranged from 0.71 to 0.81 mmol Trolox equivalents/kg.

Despite their operational simplicity and accessibility, maceration and infusion generally require relatively long processing times and provide limited control over mass-transfer conditions. Prolonged exposure to oxygen, light, or elevated temperatures may also promote lipid oxidation. These limitations explain the growing interest in co-processing and assisted technologies designed to intensify compound transfer while shortening treatment time and limiting oxidative deterioration [22,37,38,39].

#### 4.1.2. Co-Pressing/Co-Processing

Co-pressing, also referred to as co-extraction, consists of adding a flavoring material either during olive crushing (co-milling) or directly to the olive paste during malaxation. By integrating flavoring into the mechanical extraction process, this approach enables the simultaneous extraction of oil and the transfer of volatile, phenolic, and other plant-derived compounds, without requiring the prolonged post-extraction contact generally associated with infusion. Co-processing may also eliminate the clarification or filtration step required after conventional infusion. Its processing time can therefore be substantially shorter: Cecchi et al. reported approximately 1 h of contact during co-milling with fresh chili peppers, compared with 10 days for infusion with dried peppers, whereas Caponio et al. used 30 min of combined malaxation, compared with 7–15 days of infusion, depending on the spice [40,41].

In a direct comparison of infusion and combined malaxation, Caponio et al. [36] found that adding dried spices during the 30 min malaxation step at 26 °C generally promoted the recovery and preservation of phenolic compounds. Total phenolic contents were 391, 396, and 489 mg GAE/kg in oils co-malaxed with basil, chili pepper, and chili pepper–garlic, respectively, compared with 373, 380 and 393 mg GAE/kg in the corresponding infused oils. The greatest difference was therefore observed for the chili pepper–garlic formulation. In this treatment, DPPH antioxidant activity increased from 0.76 to 1.05 mmol Trolox equivalents/kg and ABTS activity from 0.84 to 1.28 mmol Trolox equivalents/kg when infusion was replaced by combined malaxation. The co-malaxed chili pepper–garlic oil also contained higher concentrations of the secoiridoid derivatives 3,4-DHPEA-EDA and p-HPEA-EDA, reaching 22.90 and 23.50 mg/kg, respectively. Overall, combined malaxation produced oils with higher total phenolic contents, stronger antioxidant activity, and lower oxidative degradation than infusion, although the magnitude of the effect varied with the spice used [36]. However, co-processing outcomes depend strongly on the botanical material and operating conditions. Ingredient species, olive cultivar, maturity, moisture content, dosage, and point of addition can differently affect phenolic content, oxidative stability, and volatile composition. Therefore, these parameters must be optimized to achieve the desired chemical and sensory properties [40,42].

#### 4.1.3. Direct Addition of Natural Extracts and Essential Oils

Direct addition involves incorporating concentrated plant extracts or essential oils into the finished olive oil at predefined concentrations, thereby facilitating dosage control and formulation standardization. Essential oils mainly contribute volatile compounds, whereas plant extracts may supply phenolics, flavonoids, and carotenoids with potential antioxidant and antimicrobial activities [43]. Singh et al. [34] reported that the essential oils added at 0.05% (*v*/*v*) significantly modified the volatile profile after 45 days of storage notably reducing (E)-2-hexenal and (E)-2-heptenal. Barreca et al. [44] similarly showed that sage, oregano, rosemary, and thyme essential oils at 0.15% helped preserve the fatty-acid composition of EVOO during photo-oxidative stress. Kalompatsios et al. [45] found that oregano essential oil at 2% provided the strongest protection against primary and secondary oxidation, producing a TOTOX value of 34.26 after 28 days, close to 29.86 for the BHT control. Rosemary at 1% also maintained substantial radical-scavenging activity, whereas some citrus-oil formulations temporarily increased peroxide or p-anisidine values. Gonçalves et al. [46] observed lower formation of oxidation-related compounds in oils flavored with basil, lemon, and chili pepper, but greater deterioration in garlic-flavored oil. Results obtained through maceration and co-crushing further confirm that botanical source, concentration, and incorporation method influence phenolic enrichment and oxidative stability [47,48]. Moreover, phenolic content is associated with antioxidant capacity, bitterness, and pungency [49], while oxygen exposure during storage may reduce phenolics and attenuate positive aromas [50]. Therefore, ingredient concentration, oil-matrix compatibility, storage stability, and consumer acceptance should be jointly optimized [51].

**Figure 1:**
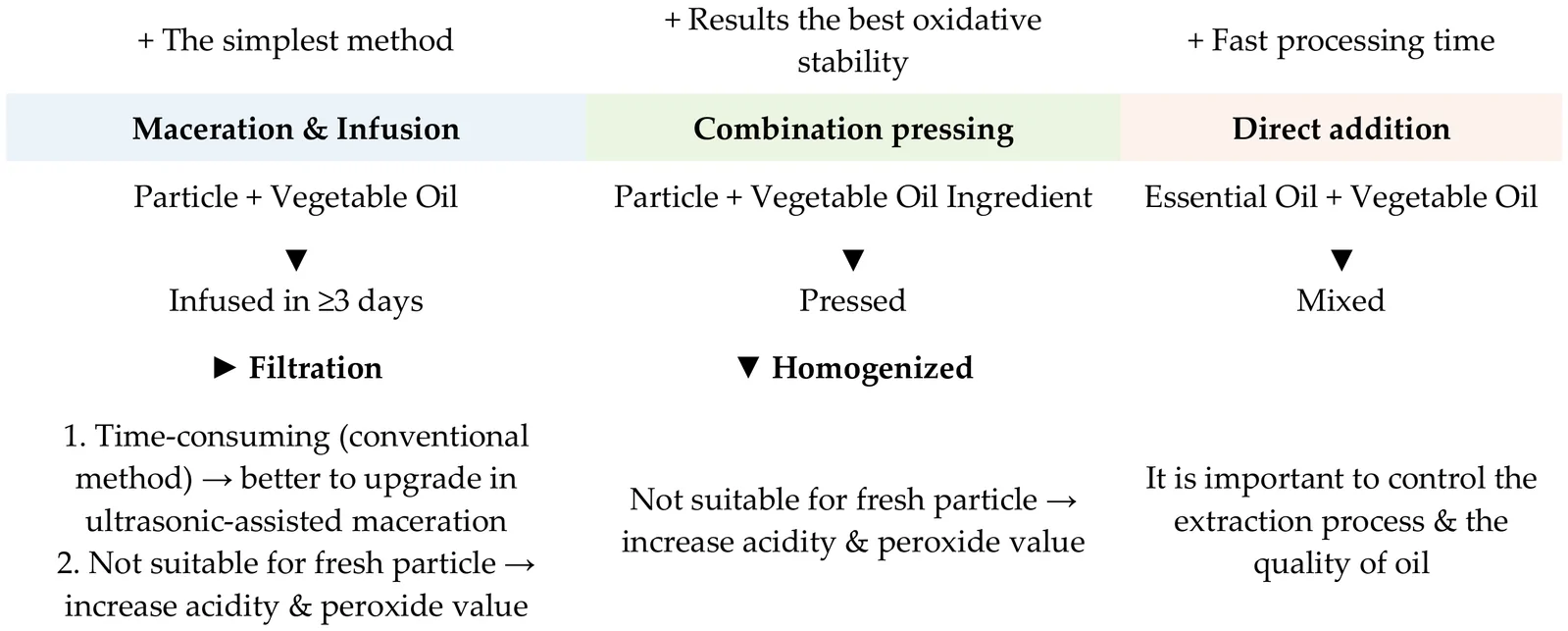
Flavored Vegetable Oil Processing Methods [52].

Flavored vegetable oil processing approaches are illustrated in Figure 1.

### 4.2. Emerging Extraction Technologies

#### 4.2.1. Ultrasonic-Assisted Extraction

Ultrasonic-assisted extraction (UAE) is considered a promising green technology for recovering bioactive compounds and producing enriched or flavored oils because it can accelerate mass transfer, reduce treatment time, and decrease solvent and energy requirements compared with conventional extraction or maceration methods [2,53]. Its mechanism is primarily based on acoustic cavitation: ultrasonic waves generate microscopic bubbles that grow and collapse, producing localized shear forces, microjets, and pressure gradients. These effects disrupt plant tissues, increase cell permeability and facilitate the release and diffusion of intracellular phenolic compounds and volatile constituents into the surrounding oil phase [54,55]. The effectiveness of UAE depends strongly on the plant material, lipid matrix, and operating conditions. Corsetti et al. [56] optimized the ultrasound-assisted flavoring of refined olive oil with Sorrento lemon peel at 15 °C for 3 min using a peel-to-oil ratio of 0.15 g/g. Under these conditions, total phenolic content increased more than threefold, from 54.24 ± 8.04 to 179.06 ± 6.87 mg GAE/kg, while the peroxide value remained approximately 3.9–4.1 meq O_2_/kg and the pre-existing off-flavors were masked. Conversely, Peres et al. [42] found that ultrasound pretreatment did not significantly modify the conventional quality parameters, total phenolic content, or pigment concentration of flavored oils obtained from low-moisture Cornicabra olives co-extracted with thyme, indicating that UAE is not systematically beneficial under all processing conditions. Treatment intensity and duration must also be carefully controlled. Using treatment times of 3–17 min and powers of 256–640 W, Kraljic et al. [57] reported a cultivar-dependent response: oil yield increased by approximately 4% in *Oblica*, phenolic content rose by up to 17% in *Istarska* Bjelica, and oxidative stability and antioxidant capacity improved by approximately 13–15% in *Istarska* Bjelica and *Levantinka*. However, prolonged treatment or excessive power decreased phenolic content, α-tocopherol concentration, antioxidant capacity, and oxidative stability in some cultivars. These findings confirm that excessive acoustic energy may promote oxidative reactions and the degradation of thermolabile or oxidation-sensitive compounds [2,57,58].

**Figure 2:**
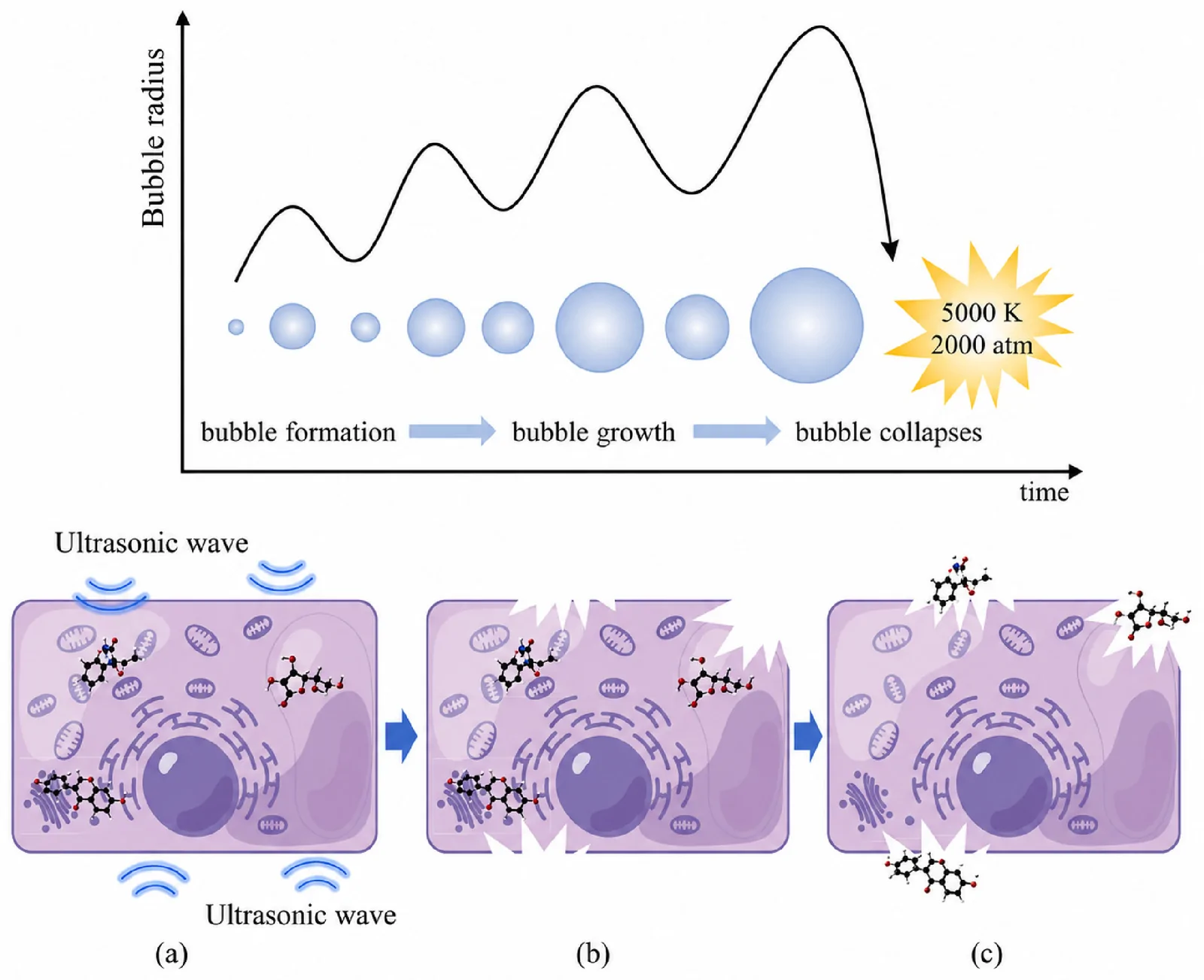
The mechanism of ultrasonic-assisted extraction. (**a**) indicates that ultrasound acts on an intact cell. (**b**) indicates cell rupture after ultrasonic treatment. (**c**) indicates that the bioactive components are released [55].

**Figure 3:**
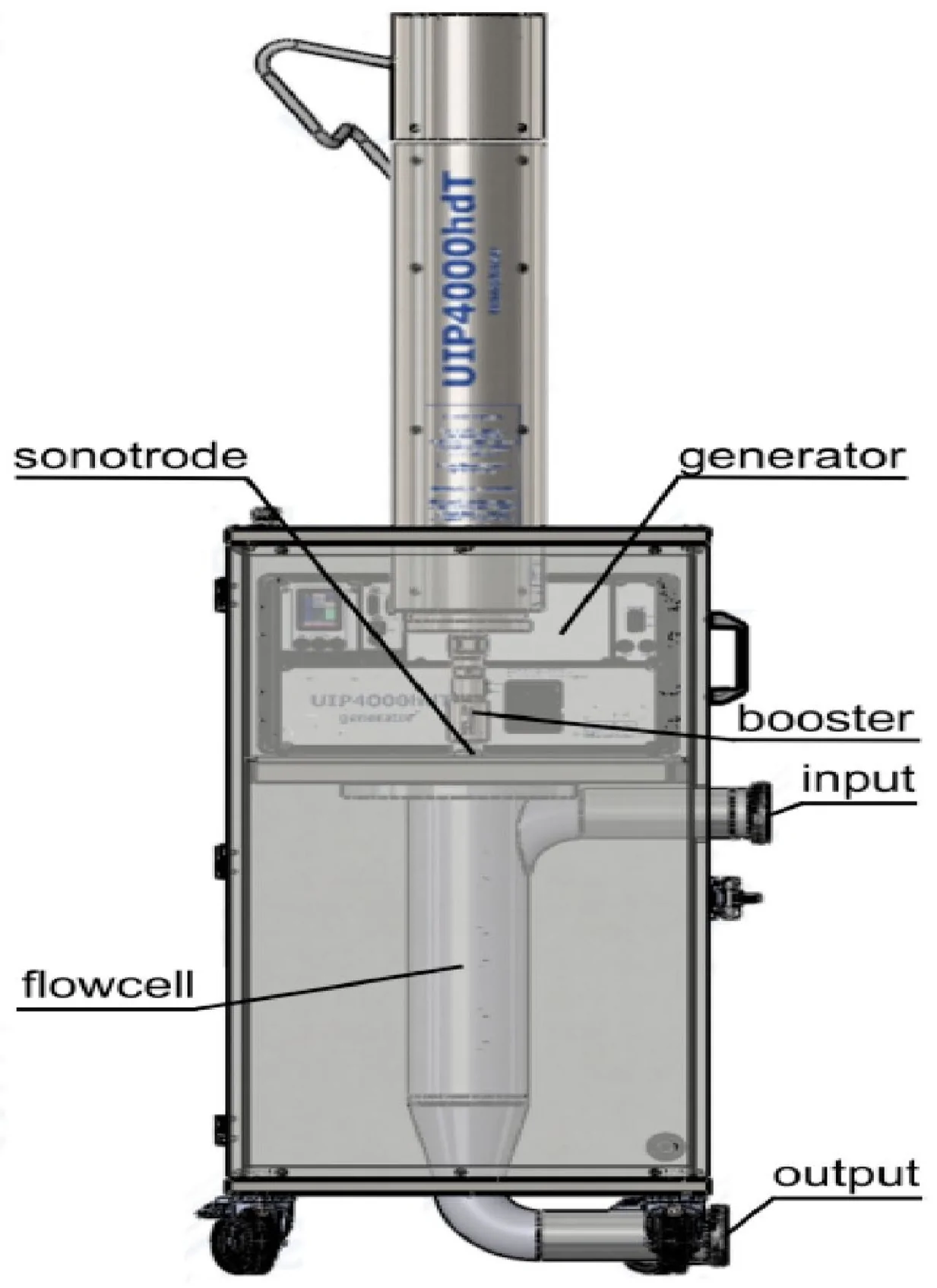
Representation of an ultrasound system used in industrial trials [62].

Although industrial and continuous-flow applications have already been demonstrated, wider implementation remains constrained by differences in equipment design, acoustic frequency, effective power, energy density, flow rate, temperature, treatment duration, and matrix properties. Industrial studies have reported oil-yield increases of 18–43% and energy-consumption reductions of approximately 35–48%, demonstrating the technological and economic potential of continuous ultrasound treatment [59]. Nevertheless, standardized reporting of operating and energy parameters is still required to enable reliable comparisons and reproducible scale-up. Further validation should therefore address cultivar- and matrix-specific optimization, equipment costs, process integration, regulatory and safety requirements, consumer acceptance, and the long-term storage stability of flavored oils [56,60,61]. The mechanism and principal effects of UAE are illustrated in Figure 2, while an industrial ultrasound system is presented in Figure 3.

#### 4.2.2. Microwave-Assisted Extraction

**Figure 4:**
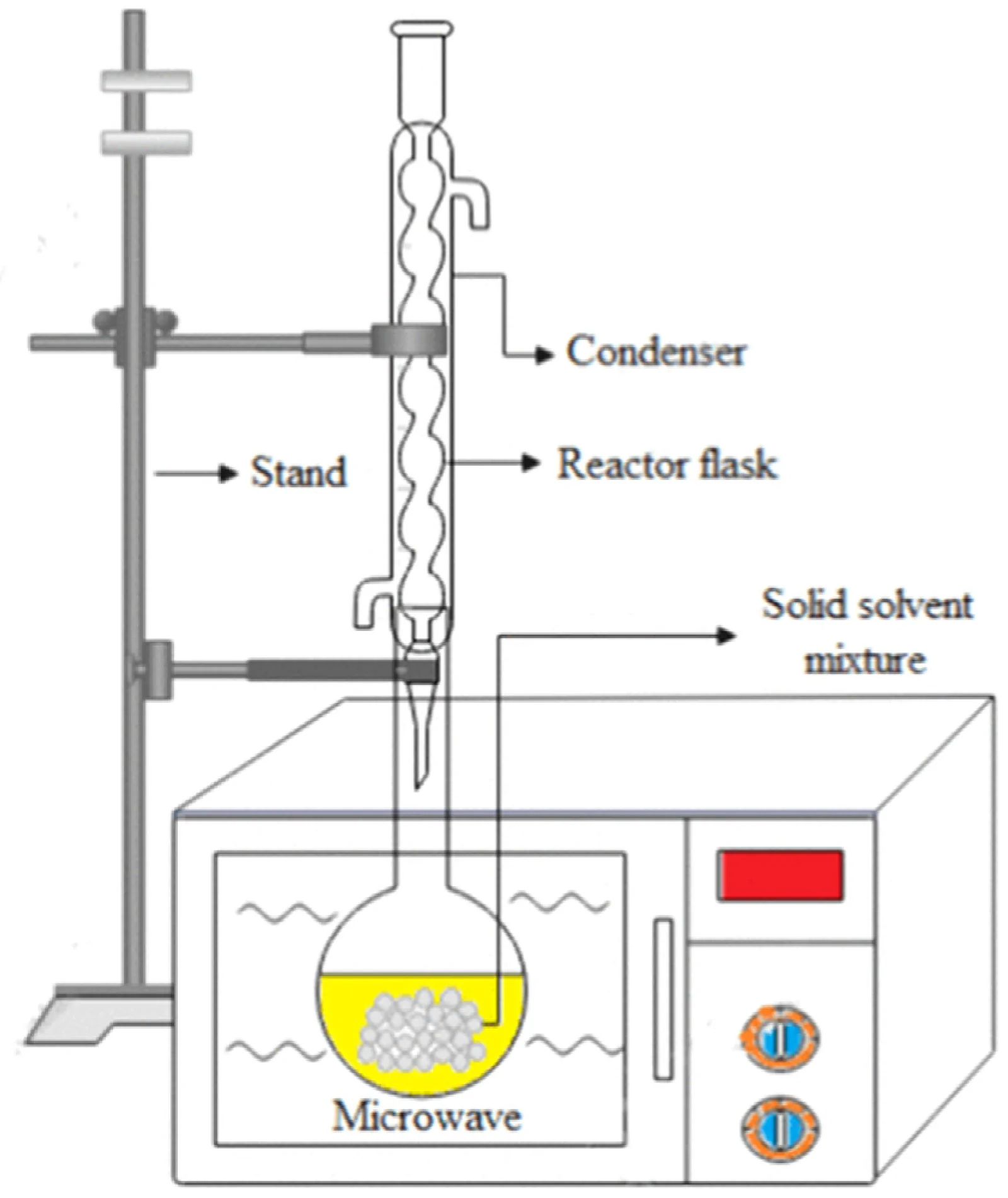
Diagram of microwave assisted extraction [16].

Microwave-assisted extraction (MAE), illustrated in Figure 4, is an emerging technology based on dielectric heating, in which microwave energy induces dipole rotation and ionic conduction, producing rapid volumetric heating. This mechanism can disrupt plant cell structures, improve solvent penetration, and accelerate the release of intracellular compounds. As a result, MAE can enhance the recovery of phenolic and other bioactive compounds while reducing processing time and, in some applications, solvent consumption [63,64]. However, the magnitude of these benefits, particularly in terms of energy savings, remains insufficiently quantified because standardized comparisons with conventional extraction methods are still limited. MAE performance is strongly influenced by microwave power, treatment time, temperature, solvent composition, and solid-to-solvent ratio. The substantial variation in these parameters among published studies complicates reproducibility and the establishment of standardized operating protocols [63]. Most investigations have been conducted at laboratory scale, whereas pilot- and industrial-scale validation remains scarce. Consequently, questions regarding scalability, process stability, energy efficiency, and economic feasibility remain unresolved [16,63]. Additional limitations include non-uniform heating, limited microwave penetration at larger sample volumes, equipment costs, and the potential degradation of thermolabile phenolic and volatile compounds under excessive power or prolonged treatment [15,65,66].

#### 4.2.3. Pulsed Electric Field Technology

**Figure 5:**
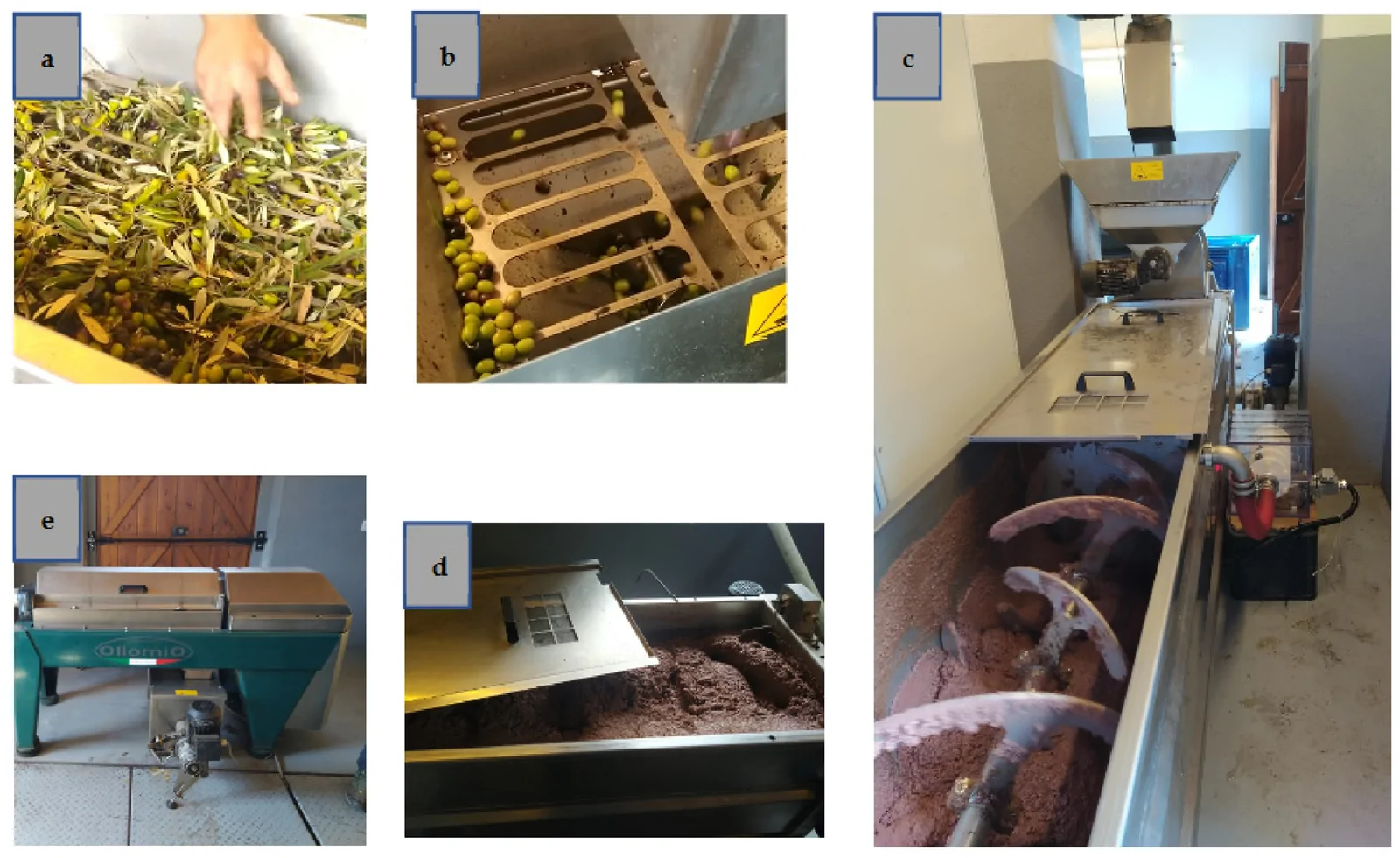
Steps of olive oil extraction line with the PEF chamber before the malaxer container. (**a**) Cleaning; (**b**) crushing; (**c**) PEF treatment; (**d**) malaxation; and (**e**) centrifugation [70].

Pulsed electric field (PEF) technology is a non-thermal treatment based on the application of short, high-voltage pulses that induce electroporation of plant cell membranes, thereby improving the release of intracellular oil and bioactive compounds during extraction (Figure 5) [14,17,67]. Compared with conventional mechanical extraction, PEF can increase oil yield while maintaining most conventional quality parameters. Martínez-Beamonte et al. [68], for example, reported a 17% increase in oil yield from Empeltre EVOO, accompanied by only minor changes in phytosterol and phenolic composition, supporting the potential of PEF to improve extraction efficiency without substantially compromising the chemical characteristics of the oil.

Similarly, Yang et al. [69] observed a 5.6% increase in oil yield from *Koroneiki* olives, together with increases of 7.6% in total phenolics, 18.3% in flavonoids, and 76% in oleuropein. The concentrations of α-, β-, γ-, and δ-tocopherols also increased by 9.8%, 10.7%, 13.6%, and 38.4%, respectively. These changes were accompanied by improved DPPH and ABTS antioxidant activities, whereas K232, K270, ΔK, and saponification values were not significantly affected.

A broader comparison by Pérez et al. [14] reported extraction yields of up to 18% for PEF-treated *Tsounati*, *Amfissis*, and *Manaki* olives, a 54% increase in yield for *Arbequina* paste treated at 2 kV/cm without malaxation, and a 40.5% reduction in residual oil losses from *Nocellara del Belice* pomace. However, the response to PEF is strongly cultivar-dependent. Montoro-Alonso et al. [17] reported marked increases in phenolic compounds in *Carolea*, *Coratina*, and *Ottobratica*, whereas the effects were less pronounced in cultivars such as *Manzanilla*, *Empeltre*, *Picholine*, and *Nocellara del Belice*. In *Koroneiki*, total phenolics, flavonoids, and oleuropein increased by 7.6%, 18.3%, and 76%, respectively.

Energy requirements also vary considerably, ranging from approximately 1.6 to 70 kJ/kg of olives, depending on field strength, pulse characteristics, cultivar, and system configuration [57,65,70]. This wide variability complicates direct comparisons of energy efficiency and highlights the need for standardized reporting of PEF operating parameters.

#### 4.2.4. High-Pressure Processing

**Figure 6:**
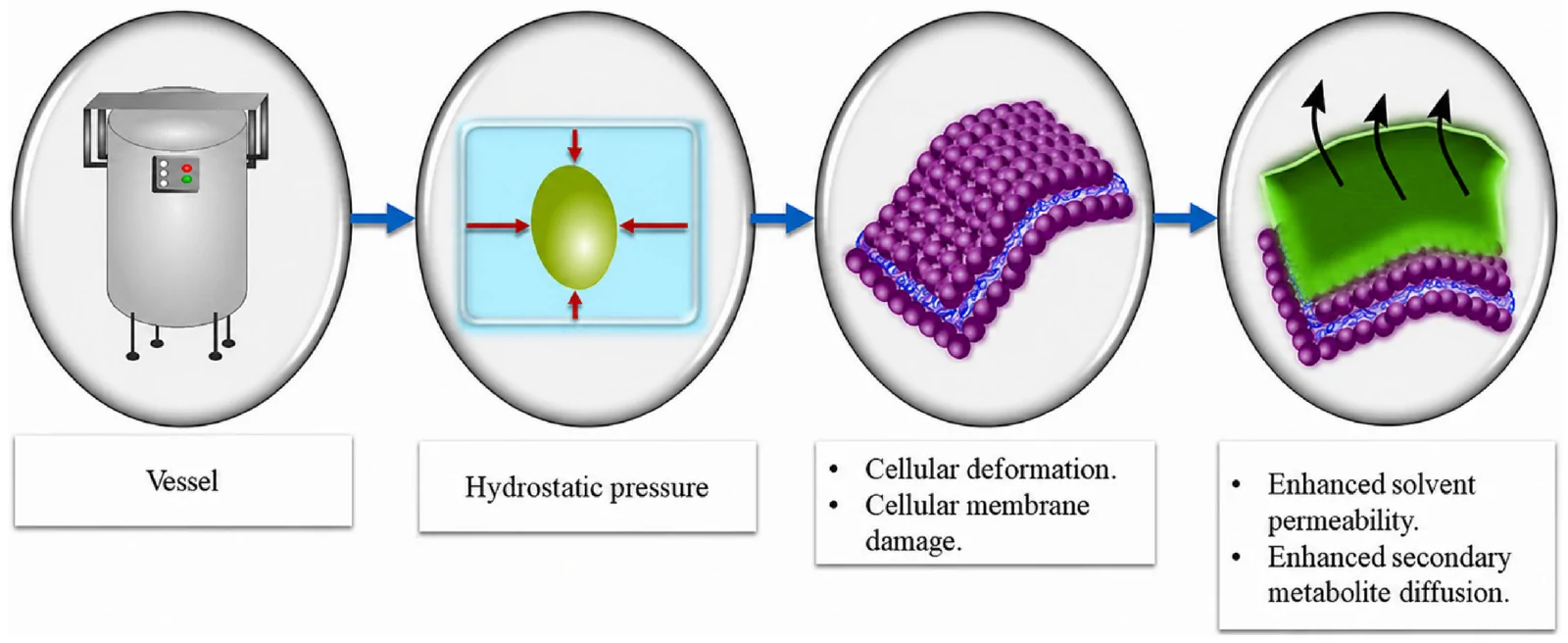
Cellular membrane deformation and damage caused by HPP [14].

High-Pressure Processing (HPP), also termed high hydrostatic pressure (HHP), is a non-thermal technology typically applied at 100 to 600 MPa for several minutes to disrupt olive tissues and facilitate the release of oil and minor bioactive compounds while minimizing thermal damage (Figure 6) [71,72,73]. Treatment of *Arbequina* olives at 300 or 600 MPa for 3–6 min increased oleocanthal and oleacein concentrations in the fruit by more than 50% and enhanced pigment extractability [13,74]. However, the resulting oils contained fewer phenolic compounds, possibly because polyphenol oxidase (PPO) and peroxidase (POX) were not completely inactivated. Conversely, α-tocopherol, squalene, and fatty-acid composition remained largely unchanged [73].

Studies on *Tsounati*, *Amfissis*, and *Manaki* olives also reported cultivar-dependent improvements in extraction yield, phenolic composition, and oxidative stability after treatments at 200–600 MPa. At 600 MPa for 5 min, HPP increased hydroxytyrosol by 44% and enhanced lignans, oleuropein, apigenin, and α-tocopherol without significantly affecting conventional quality indices. After six months, peroxide value, K232, K268, and free acidity remained stable, while several biophenols were better preserved [17]. Nevertheless, process performance depends on cultivar, pressure, treatment duration, and endogenous enzyme activity, while high equipment costs and batch operation may restrict industrial implementation [71].

#### 4.2.5. Supercritical Carbon Dioxide Extraction (Sc-CO_2_)

**Figure 7:**
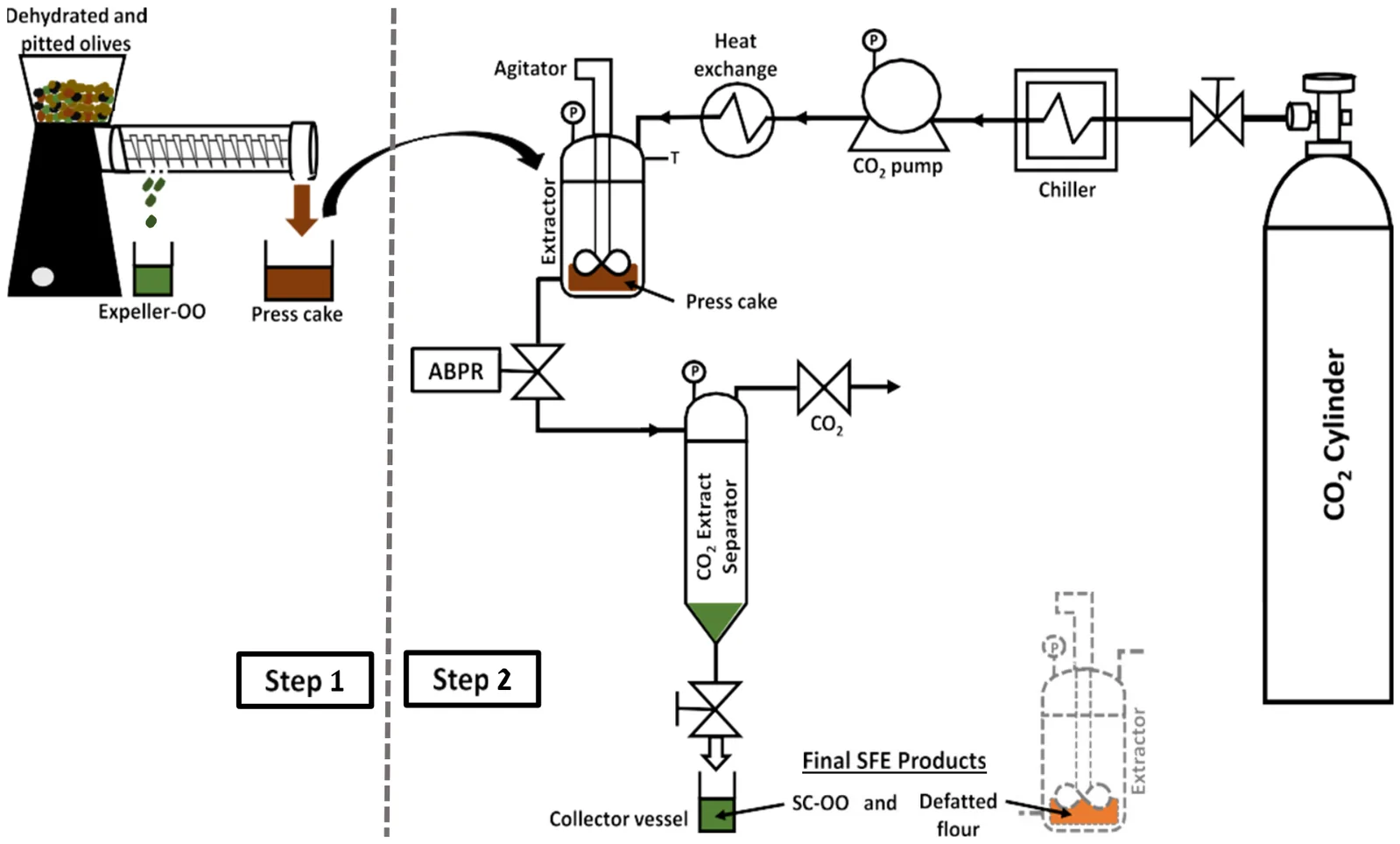
Two-step sequential extraction procedure scheme of expeller (step 1) and supercritical CO_2_ (step 2) technologies to obtain OO from dehydrated olives [19].

Supercritical carbon dioxide extraction (sc-CO_2_) is conducted above the critical temperature and pressure of CO_2_, namely 31.1 °C and 7.38 MPa, respectively (Figure 7). According to De Feo et al. [75] supercritical CO_2_ combines liquid-like density with gas-like diffusivity, low viscosity, and negligible surface tension. These properties facilitate penetration into plant matrices and the selective recovery of lipophilic constituents. Its solvating power can be adjusted by modifying pressure and temperature, while relatively low operating temperatures help limit the degradation of thermolabile compounds. Nevertheless, extraction efficiency also depends on CO_2_ flow rate, extraction time, particle size, moisture content, bed packing, and matrix characteristics.

The matrix-dependent performance of sc-CO_2_ was demonstrated by Lopes et al. [76]. These authors obtained maximum extraction yields of 14.8% from *Garcinia brasiliensis* seeds at 40 °C and 28 MPa and 15.2% from *Tabernaemontana catharinensis* seeds at 6 °C and 28 MPa. Most of the extract was recovered during the initial stage of extraction, while pressure accounted for 91.4% and 69.5% of the yield variability in the two matrices, respectively. The *G. brasiliensis* extract contained approximately 35% oleic acid and 19.6% δ-tocopherol. However, ethanol-based Soxhlet extraction produced a higher yield for *G. brasiliensis* (17.5%) but a lower yield for *T. catharinensis* (8.2%), confirming that the relative performance of Sc-CO_2_ depends on the botanical matrix and the polarity of the target compounds.

As discussed by De Feo et al. [75], the non-polar nature of pure CO_2_ makes it particularly effective for recovering lipids, terpenes, carotenoids, tocopherols, and other lipophilic constituents, but less efficient for polar phenolic compounds. Polar modifiers such as ethanol or water can improve the recovery of hydrophilic compounds, although they may increase downstream solvent separation and recovery requirements. In the extraction of green coffee compounds, Shofinita et al. [77] reported the use of Sc-CO_2_ at 35.2 MPa with 5% ethanol as a co-solvent. The authors noted that the high operating pressure increased energy requirements, whereas the co-solvent introduced additional solvent-handling and recovery steps.

Equipment configuration and process scale-up also influence extraction performance. Milovanovic et al. [78] reported laboratory-scale yields ranging from 11.9% to 18.0% for Silybum marianum seeds, depending on equipment design, and yields of up to 26% following scale-up to a semi-industrial system. For a theoretically scaled industrial system comprising two 500 L extractors and a 180 min extraction cycle, the estimated specific manufacturing cost was €35.94 kg^−1^ of extract. Utilities and fixed capital investment accounted for approximately 39% and 30% of the production costs, respectively. Consequently, Sc-CO_2_ may be suitable for producing concentrated lipophilic extracts intended for olive-oil flavoring, but its suitability should be assessed separately for each botanical material by considering extraction yield, selectivity, operating pressure, energy consumption, production scale, product value, and sensory compatibility.

## 5. Comparative Analysis of Olive Oil Flavoring Technologies

Table 3 and Table 4 summarize the main characteristics of conventional and emerging olive oil flavoring technologies, highlighting the trade-offs among extraction efficiency, preservation of bioactive compounds, oxidative stability, energy consumption, processing time, and industrial feasibility. No single technology currently satisfies all these criteria. Therefore, the selection of an appropriate approach should be based on the desired product characteristics, botanical ingredient, production scale, processing infrastructure, and economic constraints. Among conventional approaches, maceration and infusion remain attractive because of their simplicity, low capital requirements, and relatively low energy demand. However, their prolonged processing times can increase the risk of oxidative deterioration, while the use of fresh plant materials may increase free acidity and peroxide values because of their moisture content [52,65]. These methods may therefore be particularly suitable for small-scale or artisanal production, whereas their implementation in high-throughput industrial systems is more challenging.

Co-pressing (co-extraction) represents the most promising conventional technique because flavoring materials are incorporated during oil extraction, allowing the simultaneous transfer of volatile and phenolic compounds into the oil matrix. This approach has been associated with improved antioxidant activity, enhanced oxidative stability, and better integration into existing olive oil production lines. Nevertheless, its effectiveness depends on the botanical material, olive cultivar, and processing conditions, and the use of fresh ingredients may adversely affect oil quality if moisture is not adequately controlled [43,65].

Direct addition of essential oils or plant extracts provides the shortest processing time and high formulation reproducibility, allowing precise control of flavor intensity and ingredient concentration. However, high concentrations or inappropriate combinations may mask the characteristic sensory attributes of EVOO or negatively affect quality and oxidative stability. Optimization of the extract or essential-oil type, concentration, and compatibility with the oil matrix is therefore essential [15,65].

Emerging technologies generally provide greater control over compound transfer and may reduce processing time. Ultrasound-assisted extraction can accelerate mass transfer through acoustic cavitation and enhance the recovery of selected phenolic and volatile constituents [56,58,79,80]. Microwave-assisted extraction provides rapid volumetric heating but requires careful control of power, temperature, and treatment duration to limit the degradation of thermolabile compounds [63,81,82]. Pulsed electric field treatment promotes electroporation and may increase oil yield and phenolic recovery, although its effectiveness varies among cultivars and operating conditions [57,64,68,69,70,83]. High-pressure processing offers a non-thermal alternative for the release or preservation of selected bioactive compounds, but its high capital cost and predominantly batch-based operation remain significant limitations [73,84,85]. Supercritical carbon dioxide extraction enables selective recovery under relatively mild thermal conditions and avoids conventional organic solvents; however, its limited affinity for highly polar compounds, high-pressure equipment requirements, and energy demand constrain wider application [2,19,86,87].

From an industrial perspective, technology selection should consider not only extraction yield and total phenolic content but also sensory quality, oxidative stability during storage, process reproducibility, energy consumption, environmental performance, and economic feasibility [14,60,61,62,71,72,79]. The available evidence nevertheless remains difficult to compare because operating conditions and analytical methods vary considerably among studies. Standardized head-to-head investigations are therefore needed to compare compound-transfer efficiency, sensory quality, oxidative stability, safety, energy demand, process yield, and life-cycle impacts under equivalent conditions [14,15,16,17,60,61,62].

**Table 3:** Comparative analysis of olive oil flavoring technologies: from conventional methods to emerging green approaches.

Technique/Category	Polyphenol Enhancement	Oxidative Stability	Processing Time	Energy Consumption	Relative Cost	Industrial Applicability	Main Limitations	Ref
Traditional maceration and infusion	Low to moderate, depending strongly on infusion time and ingredient type	Low to moderate; fresh plant materials may increase acidity and peroxide values	Long, generally ≥3 days	Very low because the process is mainly passive	Very low	Easily scalable and simple to implement	Time-consuming; poorly suited to fresh ingredients that may promote acidity and oxidation	[15,43,65]
Combined pressing or co-pressing	Noticeable improvement in nutritional value and antioxidant activity	Generally high compared with other traditional methods	Moderate; integrated into the pressing stage	Low	Low	Easily integrated into existing pressing lines	Fresh herbs or spices may increase acidity and peroxide values	[52,65]
Direct addition of essential oils or extracts	Variable, depending on the type and concentration of the added extract	Potentially low; acid, p-anisidine, and total oxidation values may increase	Very short, generally a few minutes	Very low	Low	Simple to implement at industrial scale	May impair several oxidative quality parameters; high concentrations may also cause undesirable sensory effects	[43]
Ultrasound-assisted extraction (UAE)	Marked increase in oleuropein, tocopherols, and phenolic compounds	Generally improved, particularly during oil aromatization	Strongly reduced, from several days to a few minutes	Generally lower than conventional methods, although comparisons remain mainly qualitative	Moderate	Industrial scale-up is feasible but still limited by the lack of standardized operating parameters	No consensus regarding amplitude, power, and treatment duration; excessive power or prolonged treatment may degrade sensitive compounds	[2,53,54,59]
Microwave-assisted extraction (MAE)	Variable, depending on the matrix, solvent, and target compounds	Insufficiently quantified and highly dependent on operating conditions	Short, although treatment parameters remain poorly standardized	Often reported as reduced, but quantitative comparative data are limited	Moderate to high, depending on the equipment	Mainly validated at laboratory or pilot scale	Lack of standardized protocols; limited reproducibility; risk of overheating; insufficient energy and large-scale validation data	[15,16,63,64,66]
Pulsed electric fields (PEF)	Heterogeneous and cultivar-dependent; substantial increases have been reported for Carolea, Coratina, and Ottobratica, but limited effects for Manzanilla, Empeltre, and Nocellara del Belice	Generally reported as neutral or beneficial, although large-scale evidence remains limited	Malaxation time may be reduced by up to approximately 33%	Approximately 1.6–70 kJ/kg; the wide range indicates limited standardization	High initial investment, with potential profitability at medium or large scale	More suitable for medium- and large-scale production; equipment rental or shared use may be preferable for small facilities	Results are difficult to generalize; strong dependence on cultivar and treatment parameters; insufficient energy standardization	[17,43,70]
High-pressure processing (HPP)	Contrasting results for olive oil: secoiridoids may increase, whereas some phenolic compounds may decrease because of PPO and POX activity	Variable; lipid oxidation may increase in high-fat matrices	Short cycles, generally 3–7 min, but the process is discontinuous	High; reported values range from 2.5 to 3.2 kWh/kg and may greatly exceed those of thermal treatment	High; equipment costs may reach approximately US$2.5 million per unit	Adoption remains limited because of high capital costs and batch operation	High capital and operating costs; discontinuous process; limited suitability for certain matrices	[13,71,72,84]
Supercritical CO_2_ extraction (Sc-CO_2_)	Adjustable selectivity; highly effective for lipophilic compounds but less efficient for highly polar compounds without a co-solvent	Good preservation of thermosensitive compounds because of moderate temperatures and limited oxygen exposure	Variable, depending on pressure, temperature, CO_2_ flow rate, and matrix characteristics	CO_2_ compression is energy-intensive despite the relatively moderate operating temperatures	High because of the required high-pressure equipment	Mainly operated in batch mode; industrial scale-up remains technically complex	High investment cost; sensitivity to matrix moisture; need for qualified personnel; limited efficiency for polar compounds	[16,88,89]

**Table 4:** Comparison of the scope, compounds, technologies, and operating conditions covered by related studies/reviews.

Main Focus	Main Composition/Compounds Considered	Techniques/Processes Covered	Representative Conditions Reported	Flavoring	Emerging Technologies	Oxidative Stability	Industrial Aspects	Ref
Chemical composition of olive oil and health effects	Fatty acids, polyphenols, vitamins, minerals and other minor constituents; emphasis on phenolic antioxidants	Conventional olive-oil extraction; discussion of press, two-phase centrifugation and three-phase centrifugation	No standardized operating conditions are compared; the paper discusses three industrial-scale extraction systems and factors such as cultivar, ripeness, storage and extraction technology	No	No	Limited	Limited	[6]
Emerging technologies for EVOO production, consumer acceptance and olive-mill waste valorization	Phenolic compounds, tocopherols, chlorophylls, carotenoids, volatile compounds and conventional quality parameters	PEF, HPP, ultrasound/high-power ultrasound and microwave treatments compared with conventional EVOO extraction	Conditions vary among studies; examples include PEF at 0.7–1.3 kV/cm, energy inputs up to tens of kJ/kg, and HPP around 608 MPa for 6 min	No	Yes	Yes	Partial	[14]
Extraction of plant bioactives and enrichment of vegetable oils with natural antioxidants	Polyphenols, flavonoids, carotenoids, tocopherols, volatile compounds, essential oils and other plant antioxidants	Maceration/infusion, co-processing, essential-oil addition, extract addition, ultrasound-assisted enrichment, microwave-assisted infusion and several upstream extraction technologies	Highly method-dependent; examples include flavored oils stored for 7 months after maceration; rosemary/thyme-enriched oils heated for 24 h at 180 °C; ultrasound reduced treatment from hours/days to minutes; microwave-assisted rosemary infusion produced flavored oil within a few minutes	Yes	Yes	Yes	Partial	[15]
Advanced extraction of bioactive compounds from natural sources	Phenolics, flavonoids, alkaloids, essential oils, polysaccharides and other natural bioactives	SFE, MAE, UAE, subcritical solvent extraction, SPME and combined/green extraction techniques	Conditions depend on matrix and target compound; examples include SFE at 25 MPa, 40 °C, 30 min and MAE power in the 500–700 W range in cited case studies; solvent, temperature, time and ultrasound amplitude are treated as key variables	No	Yes	No	Yes, general	[16]
Sustainable vegetable-oil extraction and phenolic enrichment/stabilization	Phenolics, tocopherols, sterols, hydroxytyrosol, oleuropein derivatives, lignans, chlorophylls, carotenoids and volatile compounds	PEF, HHP, UAE, EAE, SWE, emulsions, microemulsions and nanoemulsions	Wide range according to cultivar/technology; PEF examples include 2–24 kV/cm and approximately 4–70 kJ/kg; HHP studies include 200–600 MPa for 1–5 min	Limited/indirect	Yes	Yes	Yes	[9,17]
Health effects and therapeutic potential of *Olea europaea*-derived secoiridoids	Oleuropein, oleocanthal, oleacein, ligstroside and related secoiridoids	Biological and pharmacological evidence; not an olive-oil processing technology review	Processing conditions are not a central subject; evidence is organized around biological mechanisms, bioavailability and therapeutic effects	No	No	No *	No	[9]
Antitumor properties of olive-derived compounds and extracts	Hydroxytyrosol, oleuropein, oleocanthal, oleacein, maslinic acid, phenolic alcohols, secoiridoids and triterpenes	Review of in vitro and in vivo biological studies	No olive-oil flavoring/extraction operating conditions are systematically compared	No	No	No *	No	[11]
Integrated comparison of conventional and emerging olive-oil flavoring technologies	Phenolics, secoiridoids, tocopherols, pigments, volatile compounds, fatty-acid profile and oxidation-related quality indicators	Maceration/infusion, co-pressing, direct addition, UAE, MAE, PEF, HPP and sc-CO_2_	Compares treatment time, temperature, pressure, electric-field intensity, energy demand and other operating parameters across conventional and emerging methods	Yes	Yes	Yes	Yes	Present review

## 6. Impact of Flavoring Technologies on Nutritional Quality and Health Properties

The nutritional quality of flavored olive oils depends on both the composition of the botanical material and the flavoring or extraction technology employed. Processing conditions influence the transfer, preservation, transformation, and stability of phenolic compounds, tocopherols, pigments, and volatile constituents, thereby affecting antioxidant capacity, oxidative stability, and sensory properties [6,7,8,9,10,11,20,21,22,23,24]. However, an increase in the concentration of a bioactive compound or in vitro antioxidant activity should not be interpreted directly as evidence of improved health effects, which must be demonstrated through appropriate bioavailability, in vivo, and clinical investigations [8,9,10,11].

Traditional flavoring techniques, including maceration, infusion, and co-processing, generally increase the concentration of selected bioactive compounds by promoting the transfer of phenolics, volatile constituents, and other phytochemicals from the botanical material into the oil matrix. Among these approaches, co-processing may provide a favorable compromise between processing time and compound transfer because the simultaneous processing of olives and flavoring materials can promote efficient incorporation of both phenolic and volatile constituents. However, the magnitude of this effect is highly dependent on the botanical material and processing conditions. Prolonged maceration and the use of fresh plant materials may increase free acidity and peroxide values because of their moisture content and the associated susceptibility to hydrolytic and oxidative reactions, potentially offsetting some of the compositional advantages of flavoring [43,52].

Emerging technologies provide additional opportunities to modulate the transfer of bioactive compounds. UAE can enhance mass transfer through acoustic cavitation and has been associated with increased recovery of phenolic compounds, secoiridoid derivatives, tocopherols, and volatile constituents, particularly when aromatic herbs such as basil and rosemary are used as flavoring materials [37,38]. However, excessive ultrasonic intensity or prolonged treatment may promote oxidation or degradation of sensitive compounds, indicating that the beneficial effects of UAE depend on appropriate optimization of the operating conditions. PEF treatment can enhance the release of intracellular compounds through electroporation of plant cell membranes, although its effects on phenolic enrichment and oil quality vary substantially among cultivars and processing conditions [17]. Microwave-assisted extraction (MAE) can similarly accelerate the release of bioactive compounds through rapid volumetric heating, but excessive microwave power or treatment duration may result in the degradation of thermolabile constituents [16,63,66].

The effects of HPP and sc-CO_2_ require particular consideration because their technological functions differ from those of conventional flavoring and extraction approaches. HPP may promote the release or preservation of selected secoiridoids, including oleocanthal and oleacein, while limiting thermal degradation. Nevertheless, pressure treatment can produce variable effects on phenolic composition depending on pressure level, treatment duration, cultivar, and the activity of endogenous oxidative enzymes [13]. Consequently, HPP should not be considered inherently protective of phenolic compounds, but rather as a technology whose effects depend on the specific processing window.

sc-CO_2_ can provide mild-temperature extraction of selected lipophilic and moderately polar constituents from botanical materials and can generate extracts without conventional organic-solvent residues [63,88,89]. Its relatively low operating temperature is advantageous for the recovery of thermolabile constituents; however, the limited solvating capacity of supercritical CO_2_ for highly polar phenolics remains an important limitation. The use of polar co-solvents such as ethanol can improve phenolic recovery but increases process complexity and may modify extraction selectivity [88]. Importantly, sc-CO_2_ is primarily an extraction technology for producing concentrated botanical extracts rather than a direct flavoring process, and the nutritional characteristics of the final flavored oil will depend on the composition and dosage of the resulting extract [76,81,82].

The available evidence indicates that flavoring primarily modifies the minor bioactive and volatile fractions of olive oil, while the magnitude and direction of these changes remain strongly dependent on the process and matrix [4,20,35,36,40,41,42,43,44,45,46,47,48,49]. Comparisons among studies are limited by differences in olive cultivar, botanical material, processing conditions, storage duration, extraction procedures, and analytical methods [14,15,16,17]. Moreover, the literature is dominated by compositional measurements and in vitro antioxidant assays, whereas evidence concerning bioavailability, in vivo efficacy, long-term safety, sensory acceptance, and industrial-scale performance remains limited [8,9,10,11,14,15,16,17].

## 7. Regulatory Framework and Labeling

### 7.1. Definition and Chemical Specifications of EVOO

EVOO is legally defined within the European Union according to specific compositional, physicochemical, and sensory requirements established by the applicable EU legislation, including Commission Delegated Regulation (EU) 2022/2104 and subsequent amendments [90]. Among the principal quality criteria, EVOO must have free acidity not exceeding 0.8%, a peroxide value not exceeding 20 mEq O_2_/kg, K232 not exceeding 2.50, and K270 not exceeding 0.22, in addition to complying with the relevant organoleptic requirements [90,91].

EVOO must be obtained directly from olives and exclusively by mechanical or other physical means under conditions that do not lead to alterations of the oil. The addition of herbs, spices, fruits, extracts, essential oils, or other flavoring materials to an already produced EVOO results in a product that no longer corresponds to the legal definition of unmodified EVOO as such. Consequently, flavored products cannot generally be marketed simply under the legal designation “extra virgin olive oil” when additional flavoring ingredients are incorporated into the product [92]. The regulatory status and appropriate designation depend on the composition and formulation of the final product and on the legislation applicable in the target market.

This distinction is particularly relevant for emerging flavoring technologies. Co-processing of botanical materials with olives and adding botanical extracts after oil extraction may result in chemically and legally different products, even when EVOO is used as the starting material. Therefore, technological innovation should be accompanied by a clear assessment of the regulatory status of the resulting product, including its composition, legal designation ingredient declaration, and applicable quality requirements [90,92].

### 7.2. Recent Revisions and Global Harmonization

The European regulatory framework for olive oils and olive-pomace oils has been updated in recent years. Commission Delegated Regulation (EU) 2022/2104 established rules concerning the marketing standards for olive oil and olive-pomace oil and replaced the corresponding provisions previously contained in Regulation (EEC) No. 2568/91. The framework has subsequently been amended, including by Commission Delegated Regulation (EU) 2024/1401 [90].

At the international level, the International Olive Council (IOC) establishes standards for olive oils and olive-pomace oils, including physicochemical and sensory criteria used for the classification and assessment of these products [93]. The European Union is a member of the IOC, and EU legislation and IOC standards are therefore closely aligned in several technical aspects, although their legal status and scope are not identical.

At the global level, olive oils are also covered by the Codex Alimentarius Standard for Named Vegetable Oils, CODEX STAN 33-1981, which establishes compositional and quality criteria for olive oils and olive-pomace oils [91]. However, international regulatory frameworks do not necessarily provide a harmonized and specific regulatory category for flavored olive oils. Differences among jurisdictions concerning product classification, permitted ingredients, analytical requirements, labeling, and the use of terms referring to olive oil quality may therefore create challenges for international commercialization.

Therefore, future regulatory developments should promote greater consistency in the classification and labeling of flavored olive oils, while maintaining a clear distinction between the legal category of olive oil used as the base material and the characteristics of the final flavored product [90,91,93].

## 8. Challenges and Future Perspectives

### 8.1. Technological and Methodological Challenges

Despite significant advances in olive oil flavoring technologies, several technological and methodological limitations continue to hinder their broader industrial implementation. One of the main challenges is the lack of standardized processing protocols. Operating parameters for UAE, MAE, PEF, HPP, and sc-CO_2_ vary considerably among studies, making direct comparisons difficult and limiting process reproducibility [14,15,16,17,55,63]. In addition, excessive treatment intensity, prolonged processing, or inadequate temperature control may accelerate lipid oxidation and promote the degradation of thermolabile phenolic and volatile compounds [57,58,65,66]. Future studies should therefore establish standardized and cultivar-specific processing conditions that maximize the transfer and preservation of desirable bioactive compounds while maintaining the physicochemical and sensory quality of the oil.

### 8.2. Industrial and Economic Challenges

The transition from laboratory and pilot-scale experiments to commercial production presents important operational and economic challenges. The composition of *herbs*, *spices*, *citrus* fruits, and other flavoring materials varies according to cultivar, geographical origin, maturity stage, and storage conditions, which can substantially affect extraction efficiency, the transfer of bioactive and volatile compounds, and the sensory consistency of the final product [15,17,52]. Advanced technologies such as HPP, PEF, and sc-CO_2_ generally require greater initial capital investment and more specialized equipment than conventional flavoring methods [14,16,71,75]. Their adoption may therefore be particularly challenging for small and medium-sized producers. Moreover, industrial feasibility depends not only on equipment cost but also on production capacity, process throughput, energy consumption, maintenance requirements, labor, raw-material utilization, and product yield. Economic assessment should consequently use standardized techno-economic approaches that consider both capital and operating expenditure and the potential added market value of the resulting flavored oils. Technologies offering higher extraction efficiency may not necessarily be economically preferable if they require excessive energy input, expensive equipment, or complex downstream processing.

### 8.3. Regulatory Challenges

Regulatory uncertainty remains another important barrier to the commercialization of flavored olive oils. Although the IOC and European Union legislation establish detailed physicochemical and sensory requirements for the different categories of olive oil, specific and harmonized standards for flavored olive oils remain limited [90,91,93]. Differences in product classification, analytical methods, quality criteria, and labeling requirements may therefore complicate market access and international commercialization. Under the current European regulatory framework, the addition or co-processing of herbs, spices, fruits, or other flavoring ingredients to olive oil may prevent the final product from being marketed solely under the designation “extra virgin olive oil” even when EVOO is used as the base oil [90,92]. Further regulatory harmonization is therefore required to establish clear product categories, quality specifications, analytical procedures, and labeling requirements for flavored olive oils.

### 8.4. Future Research Priorities

Based on the methodological, technological, nutritional, economic, and regulatory gaps identified throughout this review, future research should prioritize the following areas:

- **Process standardization and cultivar-specific optimization**: establish reproducible operating ranges for UAE, MAE, PEF, HPP, and Sc-CO_2_ according to olive cultivar, flavoring material, and targeted bioactive compounds, and evaluate hybrid processing strategies where appropriate.
- **Long-term quality and safety assessment**: conduct extended storage studies to determine changes in phenolic compounds, volatile profiles, oxidative stability, sensory quality, and microbiological safety under realistic storage conditions.
- **Bioavailability and health effects**: Complement compositional and in vitro studies with in vivo investigations and, where appropriate, human studies to determine whether technologically induced compositional changes translate into improved bioavailability and demonstrable nutritional or physiological benefits.
- **Techno-economic and environmental assessment**: perform techno-economic analyses and life-cycle assessments to quantify capital and operating costs, energy consumption, environmental impacts, and the feasibility of industrial-scale implementation.
- **Packaging and consumer acceptance**: investigate packaging systems that limit oxygen and light exposure and conduct sensory and consumer studies to determine acceptance of both flavored products and emerging processing technologies.
- **Regulatory harmonization**: develop internationally recognized analytical methods, quality criteria, labeling rules, and product classifications specifically adapted to flavored olive oils.

## 9. Conclusions

This review aimed to provide an integrated and comparative assessment of conventional and emerging technologies used for olive-oil flavoring, with particular attention to compound-transfer mechanisms, oxidative stability, nutritional quality, processing efficiency, industrial feasibility, and regulatory constraints.

The available evidence shows that conventional methods, including maceration, infusion, co-processing, and direct addition, remain attractive because of their simplicity, relatively low technological requirements, and ease of implementation. Their performance, however, depends strongly on the botanical material, moisture content, ingredient concentration, contact time, olive cultivar, and processing conditions. Co-processing generally offers a favorable compromise between processing time and the transfer of phenolic and volatile compounds, whereas prolonged maceration and the use of fresh botanical materials may increase the risk of hydrolytic and oxidative deterioration.

Emerging technologies provide greater opportunities to control and intensify compound transfer. Ultrasound-assisted extraction can accelerate mass transfer; microwave-assisted extraction enables rapid volumetric heating; pulsed electric field treatment promotes electroporation; high-pressure processing offers a non-thermal approach; and supercritical carbon dioxide extraction enables the selective recovery of predominantly lipophilic constituents. Nevertheless, none of these technologies can currently be considered universally superior. Their performance and suitability depend on the target compounds, botanical material, olive cultivar, operating conditions, production scale, energy requirements, equipment costs, and regulatory context.

The principal contribution of this review lies in integrating conventional and emerging olive-oil flavoring technologies within a common comparative framework that simultaneously considers chemical composition, volatile and phenolic transfer, oxidative stability, nutritional implications, processing conditions, scalability, economic feasibility, and regulatory requirements. This integrated assessment advances current knowledge by identifying the main process-dependent trade-offs and by demonstrating that increases in extraction yield or total phenolic content alone are insufficient indicators of overall product quality or nutritional benefit.

The analysis also reveals substantial knowledge gaps. Direct comparisons remain limited by heterogeneous experimental protocols, while most available studies are conducted at laboratory scale and emphasize compositional measurements or in vitro antioxidant assays. Standardized comparative studies, long-term storage and safety evaluations, sensory and consumer investigations, bioavailability studies, pilot- and industrial-scale validation, life-cycle assessments, and robust techno-economic analyses are therefore required.

Future progress will depend on combining process optimization with chemical characterization, sensory assessment, safety evaluation, economic analysis, and regulatory harmonization. Such an integrated approach is necessary to identify the technologies capable of supporting the reproducible, safe, sustainable, and commercially viable production of flavored olive oils.

## Figures and Tables

**Table 1:** Chemical composition of olive oil.

Component/Fraction	Main Constituent or Typical Content	Representative Compounds/Values	Ref
Major components			
Triacylglycerols (TAGs)	98–99% of olive oil	TAGs mainly esterified with oleic, palmitic and linoleic acids	[6]
Monounsaturated fatty acids (MUFAs)	55–83% oleic acid	Oleic acid (C18:1); approximately 70.6–73.8% in ‘Madural’ oils	[4,6]
Saturated fatty acids (SFAs)	7.5–20% palmitic acid	Palmitic acid (C16:0), approximately 10.0–11.7% in ‘Madural’ oil; stearic acid (C18:0), approximately 2.2–2.7%	
Polyunsaturated fatty acids (PUFAs)	2.5–21% linoleic acid	Linoleic acid (C18:2), approximately 11.6–12.3%; linolenic acid (C18:3), approximately 1.0–1.1% in ‘Madural’ oils	
Minor components			
Free fatty acids	Minor fraction	Mainly expressed as free oleic acid; acidity approximately 0.10–0.35% in the ‘Madural’ study	[6]
Phosphatides	Minor fraction; not quantitatively reported	Phospholipid fraction	
Glycerol	Minor fraction; not quantitatively reported	Free glycerol	
Hydrocarbons	Minor unsaponifiable fraction	Squalene	[9]
Sterols/phytosterols	Total sterols approximately 1893–1978 mg/kg in ‘Madural’ oils	β-Sitosterol, campesterol, stigmasterol, clerosterol, Δ5-avenasterol	[4,6,9]
β-Sitosterol	Predominant sterol	Approximately 83.4–86.3% of individual sterols in ‘Madural’ oil	[4]
Campesterol	Minor sterol	Approximately 2.2–2.4%	
Stigmasterol	Minor sterol	Approximately 0.4–0.5%	
Δ5-Avenasterol	Minor sterol	Approximately 9.8–10.7%	
Triterpene alcohols	Minor fraction	Erythrodiol and uvaol, approximately 0.8–1.5% in the studied oils	
Tocopherols (Vitamin E)	Approximately 203–376.5 mg/kg in the ‘Madural’ study	Mainly α-tocopherol; smaller amounts of β- and γ-tocopherols	
α-Tocopherol	Major tocopherol isoform	Approximately 203–369.3 mg/kg in ‘Madural’ oils	
β-Tocopherol	Minor tocopherol isoform	Generally, <1–3 mg/kg	
γ-Tocopherol	Minor tocopherol isoform	Approximately <0.1–5.6 mg/kg	
Pigments	Minor fraction	Chlorophylls and carotenoids	[6,9]
Phenolic compounds	Highly variable; approximately 50–1000 mg/kg reported across olive oils	Secoiridoids, phenolic alcohols, phenolic acids, flavonoids and lignans	
Total phenolic compounds	Approximately 200–269 mg/kg in flavored and monovarietal ‘Madural’ oils	Expressed as caffeic-acid equivalents	[4]
Phenolic alcohols	Minor phenolic subclass	Hydroxytyrosol, tyrosol	[6,9]
Phenolic acids	Minor phenolic subclass	Vanillic, gallic, coumaric, caffeic, hydroxycinnamic and hydroxybenzoic acids	
Secoiridoids	Major bioactive phenolic subclass	Oleuropein, ligstroside, oleuropein aglycone, ligstroside aglycone, oleacein and oleocanthal	
Lignans	Minor phenolic subclass	Pinoresinol, 1-acetoxypinoresinol	
Flavonoids	Minor phenolic subclass	Luteolin, apigenin	[9,17]
Volatile compounds	Minor fraction with high sensory relevance	Aldehydes, alcohols, ketones, esters and hydrocarbons	[9]
Alcohols	Part of volatile fraction	Aliphatic alcohols	
Aldehydes	Part of volatile fraction	Aliphatic aldehydes; several compounds contribute to aroma	[4,6,9]
Ketones	Minor volatile fraction	Various volatile ketones	
Waxes	Minor non-glyceride ester fraction	Approximately 31–49 mg/kg in ‘Madural’ oils	

## Data Availability

Data are contained within the article.

## Abbreviations

The following abbreviations are used in this manuscript:

3,4-DHPEA-EDA	Dialdehydic form of decarboxymethyl oleuropein aglycone (oleacein)
AAT	Alcohol Acetyltransferase
ABTS	2,2′-Azino-bis(3-ethylbenzothiazoline-6-sulfonic acid)
ADH	Alcohol Dehydrogenase
DPPH	2,2-Diphenyl-1-picrylhydrazyl
EAE	Enzyme-Assisted Extraction
EEC	European Economic Community
EU	European Union
EVOO	Extra Virgin Olive Oil
GAE	Gallic Acid Equivalents
GC–MS	Gas Chromatography–Mass Spectrometry
HHP	High Hydrostatic Pressure
HPP	High-Pressure Processing
IOC	International Olive Council
LOX	Lipoxygenases
MAE	Microwave-Assisted Extraction
MUFAs	Monounsaturated Fatty Acids
OO	Olive Oil
PEF	Pulsed Electric Field
p-HPEA-EDA	Dialdehydic form of decarboxymethyl ligstroside aglycone (oleocanthal)
POX	Peroxidase
PPO	Polyphenol Oxidase
PUFA	Polyunsaturated Fatty Acid
PV	Peroxide Value
Sc-CO_2_	Supercritical Carbon Dioxide Extraction
SFA	Saturated Fatty Acid
SFE	Supercritical Fluid Extraction
SWE	Subcritical Water Extraction
TAGs	Triacylglycerols
TOTOX	Total Oxidation
UAE	Ultrasound-Assisted Extraction
VOCs	Volatile Organic Compounds
VOO	Virgin Olive Oil
